# Leukoaraiosis Is a Chronic Atherosclerotic Disease

**DOI:** 10.1100/2012/532141

**Published:** 2012-05-15

**Authors:** Einor Ben-Assayag, Milija Mijajlovic, Shani Shenhar-Tsarfaty, Irena Bova, Ludmila Shopin, Natan M. Bornstein

**Affiliations:** ^1^Department of Neurology, Tel Aviv Sourasky Medical Center and Sackler Faculty of Medicine, Tel Aviv University, 6 Weizman Street, 64239 Tel Aviv, Israel; ^2^Neurology Clinic, Clinical Center of Serbia and School of Medicine University of Belgrade, Dr Subotica 6, 11000 Belgrade, Serbia

## Abstract

*Background and Purpose.* White matter changes (WMCs), or leukoaraiosis (LA), are associated with increased age, hypertension, diabetes mellitus, and history of stroke. Although several lines of evidence suggest a role of atherosclerosis in atherothrombotic vascular events, their involvement in LA remains to be determined. Our study examines this association in ischemic stroke patients. *Methods.* One hundred and seventy consecutive ischemic stroke or transient ischemic attack (TIA) patients were included. All patients underwent brain computed tomography (CT) with assessment of the extension and severity of WMCs, carotid arteries duplex scan with measurements of intima-media thickness (IMT) and plaques. *Results.* Seventy-two patients (42.4%) were found to have white matter lesions, of whom 28.8% had advanced LA. Mean IMT was significantly higher in patients with LA and with advanced LA (*P* = 0.002, *P* = 0.003, resp.). In addition, LA and LA severity were associated with existence of carotid plaque (*P* = 0.007, *P* = 0.004, resp.). In multivariate logistic regression analysis, including all vascular risk factors, LA was found to be associated with age and IMT. *Conclusion.* This study reinforces the tight association between LA and carotid atherosclerosis in ischemic stroke patients. We conclude that a chronic atherosclerotic disease underlies the pathophysiology of leukoaraiosis and its progression.

## 1. Objectives

Cerebral white matter changes, also known as leukoaraiosis [1] (LA), are frequently observed on computed tomography (CT) and magnetic resonance imaging (MRI) brain scans of elderly individuals [2–4]. These changes are seen on CT as bilateral patchy or diffuse areas of hypodensity with ill-defined margins or hyperintensities on T2-weighted MRI scans involving the periventricular and centrum semiovale white matter [5]. Although the pathogenesis of LA is not completely elucidated, previous studies have shown that white matter lesions are more common with increasing age, hypertension, diabetes mellitus, and where there is a history of stroke [6]. Moreover, in a large cohort of consecutive patients hospitalized for stroke or transient ischemic attack (TIA), LA predicted poor functional recovery during the first year following stroke onset [7].

Possible contributory factors include occlusive extra-cranial arterial disease, small vessel arteriosclerosis, transient decreases in local perfusion because of autoregulatory dysfunction, and microembolic disease [6]. While carotid atherosclerosis has been demonstrated to have strong relationship with stroke and small-vessel disease, its association with LA is controversial [8, 9]. Inflammatory processes are associated with the pathogenesis of atherosclerosis and cerebral small-vessel disease [10, 11] and also play a role in the response to ischemic events that result from atherosclerosis [11].

In the current study, we examined the association of carotid atherosclerosis, defined by intima-media thickness (IMT) and prevalence of plaques, with LA in ischemic stroke patients.

## 2. Patients and Methods

### 2.1. Patients

One hundred and seventy patients admitted consecutively to the Department of Emergency at the Tel-Aviv Sourasky Medical Center, Tel Aviv, Israel, with a documented event of acute ischemic stroke or transient ischemic attack (TIA) between January 2003 and April 2005 were included in the study. Stroke was diagnosed by an experienced vascular neurologist and supported by a CT scan. Exclusion criteria were stroke resulting from trauma or an invasive procedure, cerebral hemorrhage, history of malignant tumor, autoimmune disease, and coagulation disorders.

A written informed consent was obtained from all patients who were enrolled and signed by the patients or a first-degree relative in case of aphasia, as approved by the local Institutional Ethics Committee.

The stroke subtypes were classified according to the TOAST classification: large-artery atherosclerosis, cardioembolism, small-vessel occlusion, stroke of other determined etiology, and stroke of undetermined etiology [12]. Neurological impairment was assessed using the National Institutes of Health Stroke Scale (NIHSS) [13].

### 2.2. Diagnosis of Leukoaraiosis

Brain computed tomography (CT) scans were performed on all the patients within 24 hours of admission. Scanning was carried out without contrast enhancement and with 10 mm continuous slices. A single trained neurologist who was blinded to the patients' clinical status evaluated the existence, location, and size of brain infarcts on CT images. White matter lesions were defined as periventricular or subcortical, areas of decreased attenuation below that expected for normal white matter. The changes were always diffusely distributed within the white matter.

Leukoaraiosis was defined as ill-defined and moderately hypodense areas of >5 mm in any CT scan dimension [14]. Extension and severity of CT white matter changes (WMCs) were rated according to the score of Blennow et al. [15]. For the purpose of this study, we classified scans as advanced LA for Blennow score above 1 (therefore, score 0 represents no lesion, 1 is mild LA in one region-frontal, parietal, occipital; scores 2 and 3 are advanced LA in two or more regions).

### 2.3. Evaluation of Carotid Atherosclerosis

Duplex carotid ultrasonography was performed to evaluate the severity of carotid atherosclerosis. All ultrasound examinations were performed with a 128XP/10 Acuson equipped with a 7.5-MHz linear-array transducer, focus depth of 40 mm, and frame rate of 15 Hz. The intima-media thickness (IMT) was defined as the distance between the intimal-luminal interface and the medial-adventitial interface. We calculated the mean carotid artery IMT (mean IMT) by averaging the thickness at 4 sites at the far walls of both the right and left distal common carotid artery [16, 17]. The subjects were also classified according to the presence of well-defined atherosclerotic plaques. Plaques were defined as a focal structure that encroached more than 0.5 mm into the arterial lumen, or 50% of the surrounding intima-media thickness (IMT) value, or demonstrated a thickness of >1.5 mm as measured from the media-adventitia interface to the intima-lumen interface [17]. The ultrasound examination and assessments were performed by expert technician blinded to clinical and CT features.

### 2.4. Definitions of Risk Factors

Diabetes mellitus was defined as a fasting blood glucose of >126 mg/dL or the use of insulin or oral hypoglycemic agents; hypertension as intermittent blood pressure measurements of >140/90 mmHg or the use of antihypertensive medications. For individuals without a fasting lipid profile, hyperlipidemia was recorded if the diagnosis of hyperlipidemia was included in their medical records or if they were receiving lipid-lowering medication (HMG-CoA reductase inhibitors or fibrates). For individuals with valid lipid profiles, it was defined by the low-density lipoprotein (LDL) or non-high-density lipoprotein (non-HDL) cholesterol concentrations (in individuals displaying elevated triglyceride concentrations of ≥2.26 mmol/L) above the recommended levels according to the risk profile defined by the updated ATP III recommendations [18] or the intake of lipid-lowering medication; current smokers as those smoking at least 5 cigarettes per day.

### 2.5. Statistical Analysis

All continuous data were summarized and displayed as mean ± SD. Since IMT values have a non-normal distribution, a logarithmic transformation was employed, and all results expressing IMT values were back-transformed to geometric means ± SD. Demographic and clinical data between stroke patients with and without LA were compared by the *χ* ^2^ test and Student's *t*-test. Multivariate logistic regression analyses were used to assess the relationship of LA or LA severity with the following variables: IMT (bellow and above median = 0.65 mm), age, gender, smoking, diabetes mellitus, hypertension, hyperlipidemia, and medications. *P* < 0.05 was considered statistically significant. SPSS/WIN (version 15.0, SPSS INC, Chicago, IL, USA) software was used to carry out all statistical analyses.

## 3. Results

### 3.1. Patient Characteristics

**Figure 1:**
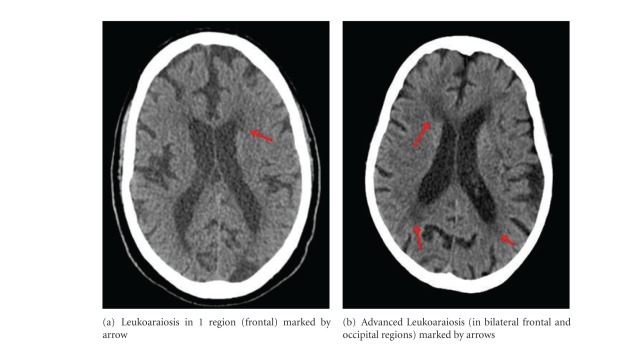
Two examples illustrating degrees of LA in CT scan.

Of the 170 acute stroke patients enrolled (mean ± SD age 66.4 ± 13.4 years, 104 men and 66 women), 94 had lacunar stroke, 32 had large-artery atherosclerotic stroke, 12 had cardioembolic stroke, 1 had stroke of undetermined etiology, and 31 had transient ischemic attack (TIA). In addition to stroke, 28.2% of the patients had diabetes mellitus, 46.7% had hyperlipidemia, 62.7% had hypertension, and 26.2% are current smokers. Seventy-two patients (42.4%) were found to have 1 or more white-matter lesions or leukoaraiosis (LA) on CT images located in frontal, parietal, or occipital region. Of the acute stroke patients, 49 patients (28.8%) were recorded to have advanced LA.  Figure 1 illustrates examples of an image from a patient suffering from LA in one region (Figure 1(a)) and a patient suffering from advanced LA (Figure 1(b)). The characteristics of stroke patients with and without LA are summarized in Table 1. Stroke patients with LA differed significantly from stroke patients without LA in their age (71.4 ± 11.3 versus 62.6 ± 13.7 years, *P* < 0.001) and the prevalence of hypertension (74.6% versus 54.1%, *P* = 0.006) and diabetes mellitus (36.1% versus 22.4%, *P* = 0.051) (Table 1).

#### 3.1.1. Relationship between LA and Carotid Atherosclerosis

We observed significantly increased mean IMT measures in stroke patients with LA compared to those without it (*P* = 0.002). Also, LA was associated with carotid plaque existence (*χ* ^2^ = 7.151, *P* = 0.013), (Table 1).

**Figure 2:**
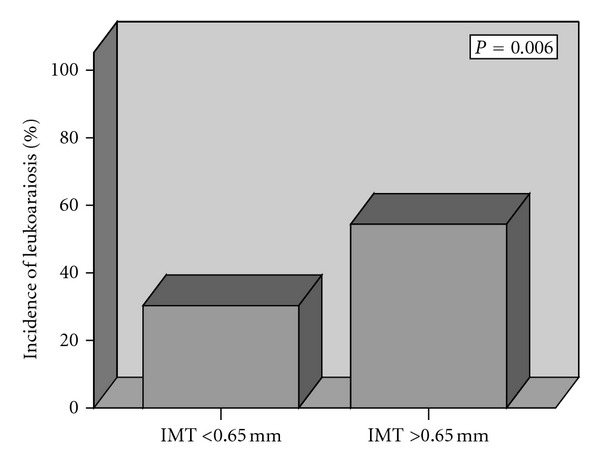
Prevalence (%) of leukoaraiosis by median of intima-media thickness (IMT). *P computed by *χ* ^2^ analysis.

We employed a logistic regression model in a stepwise forward manner. This encompassed age, gender, body mass index, use of acetylsalicylic acid, clopidogrel, beta blockers, calcium blockers, ACE inhibitors, ARBs, HMG-CoA reductase inhibitors, vascular risk factors, stroke history, stroke etiology, and mean IMT. The only two variables retained in the model for LA occurrence were IMT above 0.65 mm and age (O.R. 1.043, 95% CI 1.012–1.075, *P* = 0.006; O.R. 2.503, 95% CI 1.181–5.302, *P* = 0.017, resp.), (Table 2(a), (Figure 2).

#### 3.1.2. Relationship between Severity of WMCs and Carotid Atherosclerosis

Stroke patients with advanced WMCs represented 28.8% of the sample. Stroke patients with advanced WMCs differed significantly from stroke patients without advanced WMCs in their age (*P* < 0.001), and the prevalence of hypertension (*P* = 0.015), but not in other cardiovascular risk factors (Table 3). Measures of carotid IMT were higher in patients with advanced WMCs (*P* = 0.003) and WMCs severity was associated with carotid plaque existence (*χ* ^2^ = 8.5, *P* = 0.004), (Table 3).

In logistic regression analysis, including age, gender, body mass index, use of acetylsalicylic acid, clopidogrel, beta blockers, calcium blockers, ACE inhibitors, ARBs, HMG-CoA reductase inhibitors, and all vascular risk factors, severity of WMCs was found to be associated with age and IMT above 0.65 mm (O.R. 1.065, 95% CI 1.027–1.103, *P* < 0.001; O.R. 3.012, 95% CI 1.315–6.897, *P* = 0.009; resp.).

## 4. Discussion

This study demonstrates a significant relation between carotid atherosclerosis, reflected as IMT and existence of carotid plaques, and LA in a cohort of acute ischemic stroke and TIA patients. Previous studies have shown that white matter lesions are related to age, hypertension, diabetes, and stroke history [6]. We report that age and IMT are more significant contributors to LA compared to other cardiovascular risk factors, as seen in multivariate regression analysis, although the prevalence of hypertension and diabetes mellitus was higher in patients with LA. Additionally, stroke patients with carotid plaques tended to have more prevalence of WMCs as well as advanced WMCs than patients free of carotid plaques. Breteler et al. [19] reported that WMCs were related to atherosclerosis, defined by increased common carotid IMT and carotid plaques. Similarly, Manolio et al. [8] found that infarcts defined by MRI and white matter score were strongly associated with carotid IMT and stenosis degree after adjustment for age and sex. Furthermore, Pico et al. [20] confirmed the relationship between carotid IMT and plaques with WMCs in a 4-year follow-up study of healthy subjects.

Our study provided further evidence that the relation between carotid atherosclerosis and WMCs is independent of other vascular risk factors and history of stroke. Moreover, strengthening for those findings received by the multivariate analysis, where the findings that the only significant and independent predictors of advanced LA were age and elevated IMT as a marker of carotid atheroclerosis. It has been suggested that WMCs progress gradually over time with the accumulation of vascular risk factors [21]. Therefore our findings are in agreement with the notion that IMT and carotid plaques are a cumulative marker of cardiovascular risk. It can be assumed that patients suffering from advanced atherosclerosis (reflected as plaque formation) are in increased risk to develop advanced WMCs, based on our analysis of patients with advanced WMCs.

The present study has a limitation of possible misclassification in the diagnosis of leukoaraiosis as the diagnosis was based on CT scans rather than MR. However, the use of CT scan for the assessment of leukoaraiosis was found to be a reliable diagnostic method. Previous studies have shown a good intrareader agreement in the use of CT scan for the assessment of leukoaraiosis [22, 23]. In fact, although CT is widely recognized to be less sensitive to white-matter changes than MRI, it is still the most easily accessible neuroimaging procedure in many centers and often the only available method for brain imaging.

In summary, this study reinforces the relationship between LA and carotid atherosclerosis in a population of ischemic stroke patients, although it is not possible to suggest the probable causative relation between them. This association emerged over most vascular risk factors, as seen in multivariate regression analysis. Also, severity of WMCs was strongly related to carotid IMT and plaques. Together, these observations support diagnostic measurements of carotid IMT in stroke patients as an additional clinical tool for risk stratification. We suggest that a chronic atherosclerotic disease is probably the basic pathophysiology of leukoaraiosis and its progression.

## Figures and Tables

**Table 1:** Risk factors and clinical characteristics of stroke patients with and without leukoaraiosis.

	No Leukoaraiosis	Leukoaraiosis	
Index	(*n* = 98)	(*n* = 72)	*P*-value

Age, years	62.6 ± 13.7	71.4 ± 11.3	<0.001
Male gender, %	62.0	59.7	0.770
BMI, kg/m^2^	27 ± 4.5	26.8 ± 4.5	0.700
Diabetes mellitus, %	22.4	36.1	0.051
Hypertension, %	54.1	74.6	0.006
Hyperlipidemia, %	45.9	47.9	0.800
Current smoker, %	29.8	21.4	0.230
Ever smoker, %	56.8	53.5	0.670
Mean IMT*, mm	0.81 ± 1.12	0.86 ± 1.13	0.002
Median IMT, (IQR), mm	0.60 (0.55–0.74)	0.70 (0.6–0.84)	0.002
Carotid plaque/s, %	63.3	82.6	0.007

NIHSS	3.9 ± 4.4	4.3 ± 3.4	0.580

Medication use			

Acetylsalicylic acid, %	33.7	50.0	0.032
Clopidogrel, %	1.0	6.9	0.039
Beta Blockers, %	21.4	33.3	0.082
Calcium blockers, %	14.3	29.2	0.018
ACE Inhibitors, %	30.6	45.8	0.042
ARBs, %	1.0	4.2	0.181
HMG-CoA reductaseinhibitors, %	21.6	25.0	0.609
Within lacunar strokes, %	50.0	50.0	0.121

BMI indicates body mass index; IMT: intima-media thickness; NIHSS: National Institutes of Health Stroke Scale; ACE: angiotensin converting enzyme; ARB: angiotensin receptor blockers; HMG-CoA, 3-hydroxy-3-methylglutaryl-Coenzyme A.

*Data were logarithmically transformed before analysis, presented is the geometric mean (SD).

**Table 2:** (a) OR (95% CI) for incidence of leukoaraiosis. (b) OR (95% CI) for severity of white-matter lesions.

Multivariate logistic regression analysis		
	(a) OR (95% CI)	(b) OR (95% CI)
Adjusted for gender, BMI, ever smoking, hypertension, diabetes mellitus, hyperlipidemia, medication use, acetylsalicylic acid, clopidogrel, beta blockers, calcium blockers, ACE inhibitors, ARBs, HMG-CoA reductase inhibitors, stroke history, stroke etiology	for Leukoaraiosis occurrence	for severity of white matter lesions
Age, years	1.043 (1.012–1.075), *P* = 0.006	1.065 (1.027–1.103), *P* = 0.001
IMT above median (0.65 mm)	2.503 (1.181–5.302), *P* = 0.017	3.012 (1.315–6.897), *P* = 0.009

OR indicates odds ratio; CI: confidence interval; BMI: body mass index; ACE: angiotensin converting enzyme; ARB: angiotensin receptor blockers; HMG-CoA, 3-hydroxy-3-methylglutaryl-Coenzyme A; IMT: intima-media thickness.

**Table 3:** Risk factors and carotid ultrasonography characteristics by severity of WMCs.

Advanced WMCs			
	No	Yes	*P*-value
	(*n* = 121)	(*n* = 49)	
Age, years	63.6 ± 13.4	73.1 ± 10.8	<0.001
Male gender	62.0	59.2	0.734
BMI, kg/m^2^	27.1 ± 4.6	26.4 ± 4.1	0.373
Diabetes mellitus, %	26.4	32.7	0.415
Hypertension, %	57.0	77.1	0.015
Hyperlipidemia, %	46.3	47.9	0.848
Current smoker, %	29.1	19.1	0.192
Ever smoker, %	56.8	52.1	0.581
Mean IMT*, mm	0.81 ± 1.12	0.87 ± 1.13	0.003
Carotid plaque/s, %	64.9	87.5	0.004
Within Lacunar strokes, %	68.5	31.5	0.834

WMC indicates white matter changes; IMT: intima-media thickness.

*Data were logarithmically transformed before analysis, presented is the geometric mean (SD).
